# Plasma and Dietary Antioxidant Status as Cardiovascular Disease Risk Factors: A Review of Human Studies

**DOI:** 10.3390/nu5082969

**Published:** 2013-07-31

**Authors:** Ying Wang, Ock K. Chun, Won O. Song

**Affiliations:** 1 Epidemiology Research Program, American Cancer Society, Atlanta, GA 30303, USA; E-Mail: ying.wang@cancer.org; 2 Department of Nutritional Sciences, University of Connecticut, Storrs, CT 06269, USA; E-Mail: ock.chun@uconn.edu; 3 Department of Food Science and Human Nutrition, Michigan State University, East Lansing, MI 48824, USA

**Keywords:** antioxidant status, cardiovascular disease (CVD), human studies, review

## Abstract

Extensive evidence has demonstrated that many antioxidants such as vitamin C, vitamin E, carotenoids and polyphenols have protective effects in preventing cardiovascular disease (CVD), a chronic disease that is mediated by oxidative stress and inflammation. This review focuses on evidence from prospective cohort studies and clinical trials in regard to the associations between plasma/dietary antioxidants and cardiovascular events. Long-term, large-scale, population-based cohort studies have found that higher levels of serum albumin, bilirubin, glutathione, vitamin E, vitamin C, and carotenoids were associated with a lower risk of CVD. Evidence from the cohort studies in regard to dietary antioxidants also supported the protective effects of dietary vitamin E, vitamin C, carotenoids, and polyphenols on CVD risk. However, results from large randomized controlled trials did not support long-term use of single antioxidant supplements for CVD prevention due to their null or even adverse effects on major cardiovascular events or cancer. Diet quality indexes that consider overall diet quality rather than single nutrients have been drawing increasing attention. Cohort studies and intervention studies that focused on diet patterns such as high total antioxidant capacity have documented protective effects on CVD risk. This review provides a perspective for future studies that investigate antioxidant intake and risk of CVD.

## 1. Introduction

Cardiovascular disease (CVD) is the most common cause of death in the Western world and accounts for approximately one third of all deaths around the world. In the U.S. there were more than 80 million deaths due to CVD in 2008, accounting for one third of all deaths. Multiple factors are involved in the cause of CVD, including fixed factors (gene, age, gender), and modifiable factors (diet, smoking, environment, exercise). Formation of an atherosclerotic plaque or lesion is the common phenomenon of all types of CVD. The initiating step in the development of an atherosclerotic lesion is damage to the endothelium [1]. Oxidative stress and inflammation are key mechanistic pathways involved in endothelial dysfunction and thus atherosclerosis, which will be discussed in the following. Diet, as an important modifiable factor ameliorating CVD risk, is a health target in the public health field. This review focuses on evidence from prospective cohort studies and clinical trials in regard to the associations between plasma/dietary antioxidants and cardiovascular events.

## 2. Methods

We manually searched epidemiologic studies of antioxidants in relation to CVD risk published between 1990 and 2012 in PubMed. “Antioxidant and CVD” were used as key words for searching. Inclusion criteria included large-scale cohort studies and intervention trials in human and dietary or plasma antioxidants as exposures. Dietary antioxidants including vitamin C, vitamin E, carotenoids (especially β-carotene), polyphenols (including flavonoids and proanthocyanidins), and total antioxidant capacity (TAC) were selected because both cohort studies and intervention studies have been conducted on these antioxidants. Plasma/serum antioxidants selected as endogenous antioxidants included albumin, bilirubin, and uric acid. Vitamin C, vitamin E and carotene were selected as exogenous antioxidants. TAC is an emerging biomarker of overall antioxidant status and was also included. Eligible outcomes were CVD incidence and CVD death for albumin, bilirubin, and uric acid, vitamin C, vitamin E, and carotenoids as exposures, and CVD risk biomarkers for polyphenols and TAC as exposures due to limited studies on CVD incidence or mortality.

## 3. Key Mechanisms Involved in CVD Etiology

### 3.1. CVD and Oxidative Stress

Oxygen free radicals, also termed reactive oxygen species (ROS) [2], are molecules that contain one or more unpaired electrons and singlet oxygen. ROS are highly reactive and damaging to cells due to the unpaired electrons. The consequence is that new free radicals produced attack healthy cells and thus a chain reaction occurs. An imbalance between ROS and antioxidants in favor of the former is defined as oxidative stress [3]. Free radicals are generated through several sources, e.g., pollution, radiation, smoking, *etc*. One major endogenous source of ROS in cells is the mitochondrial electron transport chain. The inefficiency in electron transfer will cause the loss of electrons from electron transport chain complexes I (NADH-ubiquinone oxidoreductase) and III (succinate-ubiquinone oxidoreductase) [4]. These electrons reduce molecular oxygen to produce highly reactive oxygen free radicals such as superoxide anion [5]. In addition, formation of ROS can be triggered by advanced glycation end products through the nicotinamide adenine dinucleotide phosphate (NADPH) pathway [6]. Oxidative stress is one of the causative factors that have long been identified as being involved in the pathogenesis of CVD as well as many other degenerative diseases such as cancer, and immune dysfunction [7]. The adverse effects of oxidative stress on cardiovascular system are the result of endothelial dysfunction through reduction in nitric oxide (NO) availability, inflammatory response, and lipid peroxidation [8]. Recent studies have focused on the endothelium because it plays a significant role in vascular homeostasis, vascular smooth muscle proliferation, *trans*-endothelial leukocyte migration, and thrombosis and thrombolysis balance [9]. Several reviews summarized the mechanisms of oxidative stress in association with endothelial dysfunction and NO degradation, with ROS being the common mechanism by which different CVD risk factors trigger atherosclerosis [10,11]. NO is an important regulator of vascular homeostasis and cellular signaling. By reducing NO availability, ROS breaks the balance of vessel wall and results in defective endothelial-dependent vasodilation. Once formed, ROS activates nuclear factor kappa B (NF-κB) and results in transcriptional activation of over 100 genes involved in immune system and inflammatory responses such as tumor necrosis factor-alpha (TNF-α), Interleukin-1 beta (IL-1β) and IL-6 [12]. Oxidation of low-density lipoprotein (LDL) is a well-understood process contributing to the development of CVD. Oxidized LDL (oxLDL) is taken up by macrophages, followed by conversion of the macrophage into foam cells in the vascular endothelium where it contributes to the development of atherosclerotic lesion [13]. This process involves a chronic inflammatory process.

### 3.2. CVD and Inflammation

In the early stages of CVD, endothelial dysfunction triggers a chronic inflammatory process in the vessel wall [14]. Healthy endothelium does not bind to leukocytes. When the endothelial cells become inflamed, due to oxidative stress and cytokines, they express adhesion molecules that bind leukocytes [15]. Several important adhesion molecules involved in the inflammation process include intercellular adhesion molecules (ICAMs), vascular cell adhesion molecule-1 (VCAM-1), integrins, and selectins (e.g., E-selectin) [14]. Selectins play an important role in recruiting leukocytes to the injury site. They cause the leukocytes to roll along the endothelial surface. Integrins facilitate a firmer attachment of the leukocytes to the site of injury. Once adhered to the endothelium, the leukocytes penetrate into the intima and become macrophages. Monocyte chemoattractant protein-1 (MCP-1) was shown to be responsive in this process [7]. Macrophages express scavenger receptors for taking up modified lipoproteins such oxLDL, permitting them to become foam cells, which initiates the process of atherosclerosis [14].

## 4. Plasma Antioxidant Status and CVD

Antioxidants are molecules that could give electrons to oxidants thus stop the chain reactions. Human bodies are equipped with a powerful antioxidant defense system that controls deleterious reactions of ROS. This system consists of endogenous antioxidants (e.g., albumin, bilirubin, glutathione), exogenous antioxidants (e.g., tocopherols, carotenoids, vitamin C) and antioxidant enzymes (e.g., superoxide dismutase (SOD), catalase (CAT), glutathione peroxidase (GPx)), which convert substrates (superoxide anion radicals and hydrogen peroxide) to less reactive forms. Thus, plasma antioxidants that exhibit antioxidant/anti-inflammatory effects have been associated with a lower risk of CVD.

### 4.1. Albumin

Albumin is a major antioxidant in plasma, accounting for 70% of free radical-trapping activity of serum due to its thiol group and high plasma concentration [16]. In addition, the ligand-binding ability of albumin contributes to its antioxidant ability because it limits the oxidation of LDL from copper or hydroxyl radical production from iron reaction with hydroperoxide [17,18]. A meta-analysis of 8 prospective, population-based studies on albumin and coronary heart disease (CHD) found an inverse association between serum albumin concentration and CHD risk [19]. Increase in serum albumin per unit has been reported to have a 12% lower CVD incidence risk over a three-year period among the elderly [20]. The associations between low albumin and all-cause mortality have also been reported consistently in previous studies [21,22,23,24].

### 4.2. Bilirubin and Glutathione (GSH)

Bilirubin is another high-concentration protein that has antioxidant property in serum, and thus may prevent LDL from oxidation [25]. Bilirubin is the end product of heme degradation. Antioxidant function of bilirubin was commonly attributed to a redox cycle in which bilirubin is oxidized to biliverdin by lipophilic ROS and then recycled by biliverdin reductase [25]. Also bilirubin was found to have anti-inflammatory effects, through inhibiting TNF-α-induced E-selectin, ICAM-1, and VCAM-1 [26]. The Framingham Offspring Study found serum bilirubin concentration was inversely associated with CVD risk among men, but not as clearly among women [27]. A meta-analysis of 11 prospective studies found an inverse association between serum bilirubin and atherosclerosis in men, with a clear cutoff-point of ≤10 μmol/L for increased cardiovascular risk [28]. Similar to bilirubin, GSH’s antioxidant property was also attributed to the redox cycle in which GSH is oxidized by hydrophilic ROS to oxidized glutathione (GSSG). Water-soluble GSH primarily protects hydrophilic proteins from oxidation, whereas bilirubin protects lipids from oxidation [25].

### 4.3. Tocopherols

Tocopherols, forms of vitamin E, are chain-breaking lipophilic compounds that exist in human plasma and LDL in four isoforms (α-tocopherol, γ-tocopherol, β-tocopherol, and δ-tocopherol) [29]. Although γ-tocopherol is the major form of vitamin E in diet, α-tocopherol has the highest bioavailability [30]. Despite α-tocopherol has lower oxygen quenching rates than carotenoids, the higher plasma level of α-tocopherol brings its oxygen quenching capacities up to comparable magnitudes with lycopene and β-carotene [31]. Several large-scale, prospective cohort studies have focused on the relationship between plasma/serum antioxidant molecules and CVD as summarized in Table 1. The Alpha-Tocopherol, Beta-Carotene Cancer Prevention Study (ATBC) cohort consisted of 29,092 male smokers aged 50–69 years old in Finland. After a follow-up period of 19 years, men in the highest quintile of serum α-tocopherol had a 19% lower risk (relative risk (RR): 0.81; 95% confidence interval (CI): 0.75, 0.88) for death due to CVD compared with men from the first quintile [32]. Other studies did not find significant or similar results [33,34].

**Table 1:** Cohort studies on plasma levels of antioxidants and the risk for cardiovascular events ^1^.

Exposures	Study, country, year	Study population characteristics	Follow-up (year)	Outcomes	RR (highest *vs.* lowest) and 95% CI, *p-*trend
Vitamin E	NMR [33], USA 1996	725 men and women, ≥60 years	9–12	HD mortality	1.51 (0.68–3.37), 0.15
α-Tocopherol	SENECA [34], Europe 2005	1168 men and women, 70–75 years	10	CVD mortality	0.83 (0.67–1.03)
α-Tocopherol	ATBC [32], Finland 2006	29,092 male smokers, 50–69 years	19	CVD mortality	0.81 (0.75–0.88) *, <0.0001
Vitamin C	Basel [35], German 1993	2974 men	12	IHD	<22.7 (µmol/L) *vs.* higher: 1.25 (0.77–2.01), 0.38
				Stroke	<22.7 (µmol/L) *vs.* higher: 1.28 (0.40–4.09), 0.34
Vitamin C	NMR [33], USA 1996	725 men and women, ≥60 years	9–12	HD mortality	0.53 (0.27–1.06), 0.07
Vitamin C	KIHD [36], Finland 1997	1605 men, 42, 48, 54, or 60 years without CHD or ischemia	5	AMI	<11.4 (µmol/L) *vs.* higher: 2.5 (1.3–5.2) *, <0.01
Vitamin C	EPIC-Norfolk [37], UK 2001	19,496 men and women, 45–79 years	4	CVD mortality	Per increase of 20 mol/L: 0.70 (0.60–0.82) *, <0.01
Carotene	Basel [35], German 1993	2974 men	12	IHD	<0.23(µmol/L) *vs.* higher: 1.53 (1.07–2.20) *, <0.05
				Stroke	<0.23(µmol/L) *vs.* higher: 2.07 (0.78–5.46), 0.14
Total carotenoids	LRC-CPPT [38], US 1994	1883 men, 40–59 years with type II-a hyperlipidemia	13	CHD	0.64 (0.44–0.92)*, 0.01
Carotenoids	NMR [33], USA 1996	725 men and women, ≥60 years	9–12	HD mortality	0.91 (0.42–1.99), 0.68
Carotene	SENECA [34], Europe 2005	1168 men and women, 70–75 years	10	CVD mortality	0.82 (0.68–0.98) *
Uric acid	NIDDM [39], Finland 1998	1017 patients NIDDM, 45 to 64 years	7	Stroke	>295 mmol/L *vs.* lower: 1.91 (1.24–2.94) *
Uric acid	FHS [40], USA 1999	6763 men and women, mean age 47 years	117,376 person-years	CHD	M: 0.92 (0.6–1.40), >0.2; F: 1.46 (0.84–2.53), >0.2
				CVD mortality	M: 0.73 (0.52–1.02), 0.03; F: 1.25 (0.78–2.02), >0.2
Uric acid	NHANES I [41], USA 2000	5926 men and women, 25–74 years	16.4	CVD mortality	Per increase of 59.48 µmol/L:M: 1.09 (1.02–1.18) *; F: 1.26 (1.16–1.36) *
				IHD mortality	M: 1.17 (1.06–1.28) *; F: 1.30 (1.17–1.45) *
Uric acid	KIDH [42], Finland 2004	1423 men, 42, 48, 54, or 60 years	11.9	CVD death	4.77 (1.50–15.1) *, <0.05

^1^ AMI, acute myocardial infarction; ATBC, Alpha-Tocopherol, Beta-Carotene Cancer Prevention Study; CHD, coronary heart disease; CVD, cardiovascular disease; EPIC, European Prospective Investigation of Cancer and Nutrition; FHS, Framingham Heart Study; HD, heart disease; IHD, ischemic heart disease; KIHD, Kuopio Ischemic Heart Disease Risk Factor Study; LRC-CPPT, The Lipid Research Clinics Coronary Primary Prevention Trial and Follow-up Study; NHANES I, the First National Health and Nutrition Examination Survey; NIDDM, non-insulin-dependent diabetes mellitus; NMR, Noninstitutionalized Massachusetts Resident Study; SENECA, Survey in Europe on Nutrition and the Elderly, a Concerted Action; * Significant at <0.05 level.

### 4.4. Vitamin C

Known also as ascorbic acid, vitamin C works as a complementary antioxidant of carotenoids and tocopherols for quenching ROS in hydrophilic media [43]. Vitamin C and vitamin E are well known co-nutrients, as vitamin C regenerates the tocopheryl from its oxidized form. By scavenging ROS, vitamin C protects from oxidative damage by LDL and, consequently, may prevent atherogenesis [44,45]. As reported by Mezzetti *et al.* [45] in a case-control study, lower plasma vitamin C concentration was associated with higher lipid peroxidation level as measured by fluorescent products of lipid peroxidation. This inverse association was stronger in smokers than in non-smokers. Ascorbic acid may also prevent atherogenesis through interrupting inflammatory process. In a group of patients with ischemic stroke, plasma vitamin C was significantly decreased while inflammatory biomarkers CRP, ICAM-1, MCP-1, and 8-isoprostanes were significantly elevated within 2 to 5 days after stroke onset [46]. 

Vitamin C deficiency has been found to be associated with a higher myocardial infarction (MI) risk in the prospective cohort Kuopio Ischemic Heart Disease Risk Factor Study (KIHD) conducted in Finland. 1605 middle-aged men without symptomatic CHD or ischemia between 1985 and 1989 were followed for 5 years. Men with vitamin C deficiency (plasma vitamin C <11.4 µmol/L, or 2.0 mg/L) were 2.5 times as likely to have acute MI as men with higher plasma vitamin C concentrations (RR: 2.5; 95% CI: 1.3, 5.2) after adjusted for the strongest risk factors and selective dietary factors [36]. In the Basel Prospective Study conducted among 2974 men, after a follow-up of 12 years, men with low vitamin C levels (<22.7 µmol/L) had a higher risk for ischemic heart disease (IHD) (RR: 1.25; 95% CI: 0.77, 2.01) and stroke (RR: 1.28; 95% CI: 0.40, 4.09), but not statistically significant [35]. In the EPIC-Norfolk study, 19,496 men and women aged 45 to 79 years old were followed for 4 years to examine the association between plasma vitamin C level and mortality of CVD and IHD. Plasma vitamin C level was determined to be inversely associated with mortality of CVD and IHD for both men and women [37].

### 4.5. Carotenoids

Carotenoids are a widely distributed group of naturally occurring lipid-soluble pigments. They provide the red, orange, and yellow colors in fruits and vegetables. Of the 600 naturally-occurring carotenoids identified so far, only six (lutein, lycopene, zeaxanthin, β-cryptoxanthin, β-carotene, and α-carotene) represent more than 95% of total carotenoids in human plasma and associated with some health benefits [47]. Among the major carotenoids in human serum, lycopene has the highest singlet oxygen quenching rate among major carotenoids present in human serum [31]. Only α-carotene, β-carotene, and β-cryptoxanthin, but not lycopene, lutein, and zeaxanthin, have pro-vitamin A capacity.

Several observational studies have related plasma carotenoids levels with CVD risk. In a prospective nested case-control study conducted from the Women’s Health Study (WHS), women in the upper quartiles compared with the lower half of plasma lycopene had a 34% lower risk for CVD (RR: 0.66; 95% CI: 0.47, 0.95) [48]. In a European prospective study, plasma sum of concentrations of α- and β-carotenes were measured in 1168 elderly men and women [34]. After a follow-up period of 10 years, plasma carotene concentrations were associated with a lower mortality risk for CVD (RR: 0.83; 95% CI: 0.70, 1.00). In the Lipid Research Clinics Coronary Primary Prevention Trial and Follow-up Study (LRC-CPPT), after a follow-up period of 13 years, men in the highest quartile of serum carotenoids had a 36% lower risk of CHD compared with men in the bottom quartile (RR: 0.64; 95% CI: 0.44, 0.92) [38]. Men who never smoked had a more significant protective effect of plasma total carotenoids than smokers. The Basel Prospective Study investigated plasma vitamin C and carotene in relation to mortality from ischemic heart disease and stroke among 2974 men. After 12 years follow-up, men with low plasma levels of carotene (<0.23 µmol/L) had a higher risk for ischemic heart disease (RR: 1.53; 95% CI: 1.07, 2.20). Participants with both low plasma levels of carotene and vitamin C were four times as likely to die from stroke as those with normal plasma levels of carotene and vitamin C (RR: 4.17; 95% CI: 1.68, 10.33) [35]. The underlying biological mechanism by which plasma carotenoids contributed to lower CVD risk in the elderly might be related to the reduced oxidative stress and inflammation. Several cross-sectional studies have reported an inverse association between serum carotenoids levels and inflammatory biomarkers including C-reactive protein (CRP) and sICAM-1 [49,50,51]. One longitudinal study confirmed the inverse associations between serum carotenoids and inflammatory biomarkers such as leukocyte count, CRP and sICAM-1, and also found an inverse association with oxidative stress biomarker F_2_-isoprostane. Serum carotenoids were positively associated with antioxidant enzyme SOD [52].

### 4.6. Plasma Total Antioxidant Capacity (TAC) and Uric Acid

Since antioxidants work in an antioxidant network to exert protective effects, no single antioxidant could represent overall antioxidant status *in vivo*. For example, GSH regenerates ascorbic acid which then regenerates α-tocopherol from their radical forms [3,53]. Therefore, plasma antioxidant status is the result of interaction and cooperation of various antioxidants. In 1993, a new test was introduced to measure the total antioxidant status *in vivo* [54]. Then, the concept of TAC was developed and considers the synergistic role of those antioxidants rather than the simple sum of individual antioxidants [55]. Previous studies found a depletion of total antioxidant status in coronary artery disease (CAD) patients [27,56]. In contrast, an opposite result was reported in a nested case-control study that found atherosclerosis cases had significantly higher levels of serum TAC mainly due to increased uric acid levels [57]. Since plasma TAC is highly correlated with uric acid concentration, the paradoxical results for plasma TAC are rooted in the controversial findings in uric acid. Uric acid is the final oxidation product of purine catabolism, catalyzed by xanthine oxidase. Elevated uric acid level in plasma is associated with many factors such as intake of high purine foods especially animal internal organs, high fructose intake, and diuretics [58]. The physiological function of uric acid is still unclear from numerous epidemiological studies. Hyperuricemia has been found to be positively associated with CVD [39,41,42]. The Framingham Heart Study found uric acid was not associated with cardiovascular events [40]. In contrast, as an important antioxidant, uric acid was reported to be associated with a reduced rate of cognitive decline in mild cognitive impairment patients [59].

## 5. Antioxidant Intakes and CVD

Intake of fruits and vegetables has long been associated with a lower risk for several chronic diseases mediated by oxidative stress, including CVD [60]. Dietary antioxidants such as vitamin E, carotenoids, and polyphenols were thought to responsible for the cardiovascular protective effect through suppressing oxidative stress suggested by preclinical studies and epidemiological studies. However, large-scale, randomized controlled trials in human did not support this hypothesis. Although the cause of this paradox was unknown, inherent confounding in epidemiological studies and different physical conditions in study populations may partly explain it. To get some clues through comparing previous studies, evidence from prospective studies and randomized controlled trials were reviewed and summarized in Table 2 and Table 3.

**Table 2:** Cohort studies on dietary intake of antioxidants and the risk for cardiovascular events ^1^.

Exposures	Study, country, year	Study population characteristics	Follow-up (year)	Outcomes	RR (highest *vs.* lowest) and 95% CI, *p*-trend
Vitamin E: T, D, S	NHS [61], USA 1993	87,245 female nurses, 34–59 years	8	CHD (nonfatal MI + fatal CHD)	T: 0.66 (0.50–0.87) *, <0.001 D: 0.95(0.72–1.23), 0.99 S > 2 years: 0.59 (0.38–0.91) *
Vitamin E: T, D, S	HPFS [62], USA 1993	39,910 male health professionals, 40–75 years	4	CHD (nonfatal MI + fatal CHD + CABG + PTCA)	T: 0.64 (0.49–0.83) *, <0.001 D: 0.79 (0.54–1.15), 0.11 S: 0.7 (0.55–0.89) *, 0.22
Vitamin E	FMCS [63], Finland 1994	5133 men and women, 30–69 years	14	CHD mortality	M: 0.68 (0.42–1.11), <0.05 F: 0.35 (0.14–0.88) *, <0.01
Vitamin E: T	NMR [33], USA 1996	725 men and women, ≥60 years	9–12	HD mortality	0.75 (0.41–1.39), 0.40
Vitamin E: T, D, S	IWHS [64], USA 1996	34,486 postmenopausal women, 55–69 years	7	CHD mortality	T: 0.96 (0.62–1.51), 0.27 D: 0.38 (0.18–0.80) *, <0.01 S: 1.09 (0.67–1.77), 0.39
Vitamin E: T, S	HPFS [65], USA 1999	43,738 male health professionals, 40–75 years	8	Stroke (ischemic + hemorrhagic)	T: 1.25 (0.88–1.78), >0.2 S: 1.13 (0.84–1.52), >0.2
Vitamin E	Rotterdam [66], Netherland 1999	4802 men and women, 55–95 years	4	MI	1.21 (0.75, 1.98), 0.53
Vitamin E: D	ATBC [67], Finland 2000	26,593 male smokers, 50–69 years	6.1	CI	0.86 (0.70–1.06), 0.25
				SAH	0.81 (0.44–1.50), 0.55
				ICH	0.64 (0.36–1.15), 0.15
Vitamin E: S	PHS [68], USA 2002	83,639 male physicians, ≥40 years	5.5	CVD mortality	0.92 (0.70–1.21)
				CHD mortality	0.88 (0.61–1.27)
Vitamin E: D	ZES [69], Europe 2008	559 men, 65–84 years	15	CVD mortality	α-Tocopherol: 0.96 (0.82–1.12) γ-Tocopherol: 0.94 (0.79–1.12)
Vitamin E: T, S	HPFS [65], USA 1999	43,738 male health professionals, 40–75 years	8	Stroke (ischemic + hemorrhagic)	T: 0.95 (0.66–1.35), >0.2 S: 0.85 (0.59–1.24), >0.2
Vitamin C: T, D, S	HPFS [62], USA 1993	39,910 male health professionals, 40–75 years	4	CHD (nonfatal MI + fatal CHD + CABG + PTCA)	T: 1.25 (0.91–1.71), 0.98
Vitamin C	FMCS [63], Finland 1994	5133 men and women, 30–69 years	14	CHD mortality	M: 1.00 (0.68–1.45), 0.94 F: 0.49 (0.24–0.98) *, 0.06
Vitamin C: T	NMR [33], USA 1996	725 men and women, ≥60 years	9–12	HD mortality	0.38 (0.19–0.75), 0.22
Vitamin C: T, D, S	IWHS [64], USA 1996	34,486 postmenopausal women, 55–69 years	7	CHD mortality	D: 1.43 (0.75–2.70), 0.47 S: 0.74 (0.30–1.83), 0.60
Vitamin C	CWE [70], USA 1997	1843 middle-aged men	30	Stroke (nonfatal + fatal)	0.71 (0.47–1.05), 0.17
Vitamin C	Rotterdam [66], Netherland 1999	4802 men and women, 55–95 years	4	MI	1.05 (0.65, 1.67), 0.86
Vitamin C: T, S	HPFS [65], USA 1999	43,739 male health professionals, 40–75 years	8	Stroke (ischemic + hemorrhagic)	0.95 (0.66–1.35), >0.2
Vitamin C: T	ATBC [66], Finland 2000	26,593 male smokers, 50–69 years	6.1	CI	0.89 (0.72–1.09), 0.20
				SAH	1.16 (0.62–2.18), 0.61
				ICH	0.39 (0.21–0.74) *, <0.05
Vitamin C: S	PHS [68], USA 2002	83,639 male physicians, ≥40 years	5.5	CVD mortality	0.88 (0.70–1.12)
				CHD mortality	0.86 (0.63–1.18)
Vitamin C: T, D, S	NHS [71], USA 2003	85,118 female nurses, 36–63 years	16	CHD (nonfatal MI + fatal CHD)	T: 0.73 (0.57–0.94) *, <0.01 D: 0.86 (0.59–1.26), 0.52 S: 0.72 (0.61–0.86) *
Vitamin C: D	ZES [69], Europe 2008	559 men, 65–84 years	15	CVD mortality	1.02 (0.85–1.23)
Carotene	HPFS [65], USA 1993	39,910 male health professionals, 40–75 years	4	CHD (nonfatal MI + fatal CHD + CABG + PTCA)	Never smokers: 1.09 (0.66–1.79), 0.64 Former smokers: 0.60 (0.38–0.94) *, <0.05 Current smokers: 0.30 (0.11–0.82) *, <0.05
Carotene	FMCS [63], Finland 1994	5133 men and women, 30–69 years	14	CHD mortality	Null outcomes
Carotenoids: T	NMR [33], USA 1996	725 men and women, ≥60 years	9–12	HD mortality	0.64 (0.33–1.27), 0.14
Carotenoids: T, D	IWHS [64], USA 1996	34,486 postmenopausal women, 55–69 years	7	CHD mortality	T: 1.03 (0.63–1.70), 0.71 D: 1.19 (0.67–2.12), 0.89
β-Carotene	CWE, USA 1997	1843 middle-aged men	30	Stroke (nonfatal + fatal)	0.84 (0.57–1.24), 0.59
Carotenoids: D	ATBC [66], Finland 2000	26,593 male smokers, 50–69 years	6.1	CI	β-Carotene: 0.74 (0.60–0.91) *, <0.001 Lutein + zeaxantin: 0.81 (0.66–1.00), 0.10 Lycopene: 0.74 (0.59–0.92) *, <0.05
				SAH	β-Carotene: 0.67 (0.35–1.28), 0.77 Lutein + zeaxantin: 0.47 (0.24–0.93) *, <0.05 Lycopene: 0.63 (0.33–1.20), 0.13
				ICH	β-carotene: 0.66 (0.36–1.19), 0.19 Lutein + zeaxantin: 0.81 (0.46–1.43), 0.86 Lycopene: 0.45 (0.24–0.86) *, <0.05
Carotenoids	HPFS [65], USA 1999	43,740 male health professionals, 40–75 years	8	Stroke (ischemic + hemorrhagic)	β-Carotene (total) : 0.77 (0.54–1.08), >0.2 α-Carotene: 0.94 (0.66–1.34), >0.2 Lutein: 0.70 (0.49–1.01), 0.06 Lycopene: 0.96 (0.68–1.36), >0.2
β-Carotene	Rotterdam [66], Netherland 1999	4802 men and women, 55–95 years	4	MI	0.55 (0.34–0.83) *, <0.05
Carotenoids	NHS [72], USA 2003	73,286 female nurses, 34–59 years	12	CAD (nonfatal MI + fatal CAD)	α-Carotene: 0.80 (0.65–0.99) *, <0.05 β-Carotene: 0.74 (0.59–0.93) *, 0.05 Lutein + zeaxanthin: 0.90 (0.72–1.12), 0.42 Lycopene: 0.93 (0.77–1.14), 0.74 β-Crypthoxanthin: 1.17 (0.94–1.44), 0.21
Carotenoids	ZES [69], Europe 2008	559 men, 65–84 years	15	CVD mortality	α-Carotene: 0.81 (0.66–0.99) * β-Carotene: 0.80 (0.66–0.97 ) * Lutein: 0.95 (0.81–1.12) Lycopene: 0.91 (0.76–1.08) β-Crypthoxanthin: 0.86 (0.72–1.03) Zeaxanthin: 0.88 (0.70–1.10)
Flavonols and flavones	ATBC [67], Finland 2000	26,593 male smokers, 50–69 years	6.1	CI	0.98 (0.80–1.21), 0.81
				SAH	0.75 (0.40–1.41), 0.39
				ICH	0.88 (0.50–1.57), 0.41
Flavonols, flavones	WHS [73], USA 2003	38,445 women, ≥45years	6.9	CVD	No association
Flavonols, flavones	NHS [74], USA 2007	66,360 female nurses	12	Nonfatal MI	No association
				Fatal CHD	Kaempferol: 0.66 (0.48–0.93) *, <0.05
Total flavonoids and 7 subclasses	IWHS [75], USA 2007	34,489 postmenopausal women	16	Stroke mortality	No association
				CHD mortality	Anthocyanidins: 0.88 (0.78–0.99) *, <0.05 Flavanones: 0.78 (0.65–0.94) *, <0.05
				CVD mortality	Anthocyanidins: 0.91 (0.83–0.99) *, <0.05
Total flavonols, and 5 subclasses	KIHD [76], Finland 2008	1950 eastern Finnish men 42–60 years	15.2	Ischemic stroke	Flavonols: 0.55 (0.31–0.99) *, <0.05
				CVD mortality	No association
Total flavonoids and 7 subclasses	CPS-II Nutrition Cohort [77], USA 2012	38,180 men and 60,289 women, mean age 70 and 69 years	7	CVD mortality	Total: 0.82 (0.73–0.92) *, <0.05 Anthocyanidins: 0.86 (0.76–0.97) *, <0.05 Flavan-3-ols: 0.83 (0.74–0.93) *, <0.05 Flavones: 0.82 (0.72–0.92) *, <0.01 Flavonols: 0.84 (0.75–0.94) *, <0.05 Proanthocyanidins: 0.87 (0.77–0.98) *, <0.05
TAC by TEAC assay	EPICOR [78], Italy 2011	41,620 men and women	7.9	Ischemic stroke	0.41 (0.23–0.74) *, <0.01
				Hemorrhagic stroke	1.49 (0.57–3.85), 0.45
TAC by ORAC assay	SMC [79], 2012	31,035 women CVD-free ,49–83 years	12	Total stroke (CI + hemorrhagic stroke)	0.83 (0.70–0.99) *, <0.05
		5680 women with CVD history, 49–83 years	12	Hemorrhagic stroke	0.54 (0.32–0.93) *, <0.05
TAC by ORAC assay	SMC [80], 2012	32,561 women, 49–83 years	10	MI	0.80 (0.67–0.97) *, <0.05

^1^ ATBC, Alpha-Tocopherol, Beta-Carotene Cancer Prevention Study; CABG, coronary-artery bypass grafting; CHD, coronary heart disease; CI, cerebral infarction; CPS-II, Cancer Prevention Study II; CWE, Chicago Western Electric; D, diet; F, females; FMCS, Finnish Mobile Clinic Study; HPFS, Health Professionals’ Follow-up Study; ICH, intracerebral hemorrhage; IWHS, Iowa Women’s Health Study; KIHD, Kuopio Ischemic Heart Disease Risk Factor Study; M, males; MI, myocardial infarction; NHS, Nurses’ Health Study; ORAC, oxygen radical absorbance capacity; PHS, Physicians’ Health Study; PTCA, percutaneous transluminal coronary angioplasty; S, supplement; SAH, subarachnoid hemorrhage, SMC, Swedish Mammography Cohort; T, total; TAC, total antioxidant capacity; TEAC, trolox equivalent antioxidant capacity; WHS, Women’s Health Study; ZES, Zutphen Elderly Study; * Significant at <0.05 level.

**Table 3:** Randomized controlled trials of supplemental intake of antioxidants and the risk for cardiovascular events ^1^.

Treatment, dose	Study name	Follow-up (year)	Study population characteristics	Type	Outcomes	RR (treatment *vs.* placebo) and 95% CI
Vitamin E, 800 or 400 UI/day	CHAOS [81], 1996	1.4	2002 men and women, mean age 62 years, with coronary disease	Secondary	Nonfatal MI + CVD mortality	0.53 (0.34–0.83) *
Vitamin E, 50 mg/day	ATBC [82], Finland 1997	5.3	1862 male smokers with MI history	Secondary	Nonfatal MI + fatal CHD	Null outcome
					Nonfatal MI	0.62 (0.41–0.96) *
					Fatal CHD	Null outcome
Vitamin E, 50 mg/day	ATBC [83], Finland 2000	6	28,519 male smokers with no history of stroke	Primary	Stoke (SH, IH, CI) incidence and mortality	SH mortality: 2.81 (1.37–5.79) * CI incidence: 0.86 (0.75–0.99) *
Vitamin E, 400 IU/day	HOPE [84], Canada 2000	4.5	9541 men and women, ≥55 years, at high risk of CVD	Primary and Secondary	MI + stroke + CVD mortality	Null outcome
Vitamin E, 800 IU/day	SPACE [85], Israel 2000	2	196 hemodialysis patients, 40–75 years, with CVD	Secondary	MI+ ischemic stroke + PVD + unstable angina	0.46 (0.27–0.78) *
Vitamin E, 400 IU/day	MICRO-HOPE [86], Canada 2002	4.5	3654 men and women, mean age 65 years, with diabetes	Secondary	MI + stroke + CVD mortality	Null outcome
Vitamin E, 400 IU/day	HOPE [87], Canada 2005	7	9541 men and women, ≥55 years, at high risk of CVD	Primary and Secondary	MI + stroke + CVD mortality	Null outcome
	HOPE-TOO [87], Canada 2005	7.2	7030 men and women, ≥55 years, at high risk of CVD	Primary and Secondary	MI + stroke + CVD mortality	Null outcome
					Heart failure	1.13 (1.01–1.26) *
Vitamin E, 600 IU every other day	WHS [88], USA 2005	10.1	39,876 women ≥45 years	Primary	Nonfatal MI or nonfatal stroke	Null outcome
					CVD mortality	CVD mortality: 0.76 (0.59–0.98) *
Vitamin E, 600 IU every other day	WAC [89], USA 2007	9.4	8171 female health professionals, ≥45 years, with CVD history or at high risk	Primary and Secondary	MI + stroke + RP + CVD mortality	Null outcome
Vitamin E, 400 IU every other day	PHS-II [90], USA 2008	8	14,641 male physicians, ≥50 years, with or without CVD	Primary and Secondary	MI, stroke, CVD mortality	Hemorrhagic stroke: 1.74 (1.04–2.91) *
Vitamin E, 400 IU/day	[91], Israel 2008	1.5	1434 men and women, ≥55 years, diabetic with the Hp 2-2 genotype	Primary	MI, Stroke, or CVD mortality	Hp 2-2 Placebo *vs.* Hp 2-1: 2.3 (1.4–3.9) * Hp 2-2 vitamin E *vs.* Hp 2-1: 1.1 (0.6–2.0)
Vitamin C, 500 mg/day	WAC [89], USA 2007	9.4	8171 female health professionals, ≥45 years, with a history of CVD or 3 or more CVD risk factors	Primary and Secondary	MI + stroke + RP + CVD mortality	Null outcome
Vitamin C, 500 mg/day	PHS-II [90], USA 2008	8	14,641 male physicians, ≥50 years, with or without CVD	Primary and Secondary	MI, stroke, CVD mortality	Null outcome
β-Carotene, 50 mg every other day	PHS [92], USA 1996	12	22,071 male physicians, 40–84 years	Primary	MI, stroke, CVD mortality	Null outcome
β-Carotene, 30 mg/day + and 25,000 IU of retinol	CARET [93], USA 1996	4	18,314 smokers, former smokers, and workers exposed to asbestos	Primary	CVD mortality	Null outcome
β-Carotene, 20 mg/day	ATBC [82], Finland 1997	5.3	1862 male smokers with MI history	Secondary	Nonfatal MI + fatal CHD	Null outcome
					Nonfatal MI	Null outcome
					Fatal CHD	1.75 (1.16–2.64) *
β-Carotene, 50 mg every other day	WHS [94], USA 1999	4.1	39,876 women, ≥45 years	Primary	MI, stroke, or CVD mortality	Null outcome
β-Carotene, 20 mg/day	ATBC [83], Finland 2000	6	28,519 male smokers with no history of stroke	Primary	Stoke (SH, IH, CI)	IH incidence: 1.62 (1.10–2.36) *
β-Carotene, 50 mg every other day	WAC [89], USA 2007	9.4	8171 female health professionals, ≥45 years, with a history of CVD or 3 or more CVD risk factors	Primary and Secondary	MI + stroke + RP + CVD mortality	Null outcome

^1^ ATBC, Alpha-Tocopherol, Beta-Carotene Cancer Prevention Study; CARET, Carotene and Retinol Efficacy Trial; CHAOS, Cambridge Heart Antioxidant Study; CHD, coronary heart disease; CI, cerebral infarction; IH, intracerebral hemorrhage; MI, myocardial infarction; HOPE, Heart Outcomes Prevention Evaluation Study; MICRO-HOPE, Microalbuminuria Cardiovascular Renal Outcomes; PHS, Physicians’ Health Study; PVD, peripheral vascular disease; RP, revascularization procedure; SH, subarachnoid hemorrhage; SPACE, secondary prevention with antioxidants of cardiovascular disease in endstage renal disease; WAC, Women’s Antioxidant Cardiovascular Study; WHS, Women’s Health Study; * Significant at <0.05 level.

### 5.1. Dietary Vitamin E

The effects of dietary vitamin E intake on CVD risk have been investigated by several large cohort studies. An inverse association between dietary total vitamin E intake and heart disease risk was reported by several studies [61,62,64], though, findings were controversial when a vitamin E supplement was used. The Nurses’ Health Study (NHS) conducted in 1980 involved more than 87,000 U.S. female nurses between 34 and 59 years old with no history of CVD to investigate the association between dietary vitamin E intake and CVD risk. After an 8-year follow-up, women in the top quartile of total vitamin E intake had a 34% lower risk (RR: 0.66; 95% CI: 0.50, 0.87) of coronary disease compared with those in the bottom quintile. Further analysis from the study showed that the inverse association was only attributable to vitamin E supplement intake rather than vitamin E from diet. Women who took vitamin E supplements for more than two years had a 41% lower risk of coronary disease compared with their counterparts, even after adjusted for vitamin C and carotene intakes [61]. Similarly, the Health Professionals Follow-Up Study (HPFS) found higher total vitamin E intake and ≥100 IU/day vitamin E supplement use were inversely associated with CHD in men [62]. Since 1986, 39,910 U.S. male health professionals 40 to 75 years of age who were free of CHD, diabetes, and hypercholesterolemia had been followed for four years. Men in the top quintile of total vitamin E intake had a 40% lower risk (RR: 0.60; 95% CI: 0.44, 0.81) of CHD compared with those in the bottom quintile after controlling for other confounders. It was not vitamin E from diet but from supplements that appeared to be responsible for the protective effects [62]. However, analyses from the same cohort reported 6 years later revealed that vitamin E intake was not associated with stroke incidence [65]. The Iowa Women’s Health Study (IWHS) found vitamin E from diet but not from supplement was inversely associated with CHD mortality. In 1986, 34,486 postmenopausal women without CVD were recruited to investigate dietary antioxidant vitamins and CHD mortality. After an 8 year follow-up, total vitamin E intake and vitamin E from supplements were not associated with risk of CHD mortality, but vitamin E from food was inversely associated with CHD mortality (*p*_trend_ = 0.004), with women in the highest quintile of vitamin E intake having a 62% lower risk (RR: 0.38; 95% CI: 0.18, 0.80) of CHD compared with those in the lowest quintile [64]. The Finnish Mobile Clinic Study (FMCS) conducted in Finland is a population-based, large cohort study. A total of 5133 men and women were followed for 14 years and vitamin E intake was inversely associated with CHD mortality in both men and women [63]. These studies support that high vitamin E intake (either from diet or supplement) may reduce the risk of CVD. A recent study conducted in Europe distinguished α- and γ-tocopherols, however, did not observe any associations between either one with CVD mortality [69].

### 5.2. Dietary Vitamin C

Although studies on plasma vitamin C were limited, dietary vitamin C intake has been widely studied in relation to CVD risk in several prospective cohort studies. Three out of eleven studies identified found significant protective association between dietary vitamin C intake and CVD outcomes. The most significant result was observed in the NHS, in which 85,118 nurses were followed for 16 years since 1980 [71]. Women in the top quartile of total vitamin C intake had a 27% lower risk (RR: 0.73; 95% CI: 0.57, 0.94) of nonfatal MI and fatal CHD compared with those in the bottom quintile. Further analysis from the study showed that the inverse association was only attributable to vitamin C supplement intake rather than vitamin C from diet. Women who took vitamin C supplement for 2 to 4 years had a 23% lower risk of nonfatal MI and fatal CHD compared with supplement non-users [71]. Knekt *et al.* [63] also found a lower risk for CHD mortality among women in the highest category of dietary vitamin C intake compared with the women in the lowest category (RR: 0.42; 95% CI: 0.24, 0.98) in the FMCS cohort. No association was found in men. Even though vitamin E intake had no association with stroke risk in the ATBC study, higher vitamin C was associated with a lower of intracerebral hemorrhage among the male smokers at study baseline [67].

### 5.3. Dietary Carotenoids

Among the cohort studies of dietary carotenoids, seven out of ten studies reported significant associations between dietary carotenoids and CVD outcomes in a protective direction, especially for dietary β-carotene intake. In the NHS cohort, 73,286 women had been followed up for 12 years since 1984. Osganian *et al.* [72] reported inverse associations between intakes of α-carotene, β-carotene and CAD, and no associations between intakes of lutein/zeaxanthin, lycopene, or β-cryptoxanthin and CAD. In the HPFS, smoking status was a significant effect modifier in the association between carotene intake and CHD. In current male smokers, men in the highest quintile of carotene intake had a 70% (RR: 0.30; 95% CI: 0.11, 0.82) lower risk for coronary disease compared with men in the lowest quintile; male former smokers in the highest quintile had a 40% (RR: 0.60; 95% CI: 0.38, 0.94) lower risk compared with men in the first quintile. However, no associations were found among those who never smoked [62]. In the same cohort, four forms of dietary carotenoids, α-carotene, β-carotene, lutein, and lycopene were investigated for their associations with stroke after a follow-up period of 8 years. Only lutein intake was inversely associated with risk of ischemic stroke when the highest quintile was compared with the bottom (RR: 0.63; 95% CI: 0.40, 0.99). Further analysis of supplement revealed that β-carotene supplement use was not associated with risk of stroke [65].

### 5.4. Antioxidant Vitamin Supplements

Previously reviewed large cohort studies have suggested that antioxidant vitamins, especially vitamin E, vitamin C, and β-carotene may reduce CVD risk. Accordingly, these vitamin supplements were widely tested in several large-scale, randomized controlled trials to investigate their protective effects against CVD risk. However, surprisingly null or even adverse results have been reported as summarized in Table 3.

#### 5.4.1. Randomized Controlled Trials of Vitamin E

Null results on combined CVD endpoints were reported by several primary or secondary prevention trials [84,86,87,88,89,95,96]. For example, the WAC tested 600 IU vitamin E every other day among 8171 women with a history of CVD or at high risk of CVD for 9.4 years and reported no overall effect on combined cardiovascular events [89]. Three studies, including the Cambridge Heart Antioxidant Study (CHAOS) [81] and Secondary Prevention with Antioxidants of Cardiovascular Disease in Endstage Renal Disease (SPACE) [85], reported protective effects of vitamin E supplementation on combined CVD endpoints. Adverse effects of vitamin E supplementation were reported in three studies including the recently published PHS-II. In this study, 14,641 middle-aged or older male physicians with or without a baseline history of CVD were followed for 8 years. Supplement of 400 IU vitamin E every other day had no effects on major cardiovascular events and even increased hemorrhagic stroke by 74% (RR:1.74; 95%CI: 1.04, 2.91) [90]. Adverse effects of 50 mg vitamin E supplement on hemorrhagic stroke was also reported by the ATBC Study conducted in Finland [83]. The HOPE TOO study also reported a significantly increased risk of heart failure among 7030 men and women at a high risk of CVD [87]. The underlying mechanisms of the adverse effects of vitamin E supplementation are still not well known. The populations in the ABTC and PHS-II were relatively at a lower risk for CVD compared with those in other secondary prevention studies, though participants in the ATBC were male smokers, and the PHS-II included a small portion of participants with a history of CVD at baseline. The doses used in these two studies were not the highest among the vitamin E intervention trials. Recently, Milman *et al.* [91] pointed out that vitamin E supplementation might only benefit subgroups with increased oxidative stress. They conducted a prospective, double-blind, placebo-controlled trial of vitamin E supplementation (400 IU/day) among 1434 men and women of 55 years or older, diabetic and with the Hp 2-2 genotype who were susceptible to oxidative stress, and found vitamin E supplementation of 18 months significantly reduced the primary composite outcome compared with the placebo [91]. Considering genotype as a cofounder or design factor for controlling physical conditions might be a promising way in future epidemiological studies that investigate nutrition and CVD relationship.

#### 5.4.2. Randomized Controlled Trials of Vitamin C

The WAC and the PHS II are the first two large-scale, long-term trials that tested vitamin C supplementation separately from other antioxidant supplements [89,90]. Both of them reported null outcomes. The WAC was the first randomized trial that tested the effect of vitamin C supplementation (500 mg/day) among women at high risk of CVD and it found no effect of vitamin C on combined cardiovascular events [89]. The PHS II Randomized Controlled Trial was the first randomized trial that tested the effect of vitamin C supplement individually (500 mg/day) among men on CVD prevention. Similarly, no effect of vitamin C on major cardiovascular events was found in this population [90]. Other trials, in which the effect of vitamin C on CVD was investigated as part of an antioxidant cocktail, reported inconsistent results [97,98,99].

#### 5.4.3. Randomized Controlled Trials of β-Carotene

No beneficial effect of β-carotene has been documented in any large-scale, randomized controlled trials listed in Table 3. The PHS that was conducted among 22,071 male physicians, 40 years of age or older tested the effect of 50 mg β-carotene on the incidence of MI, stroke, or CVD mortality and found no significant results after 12 years of supplementation [92]. In the WHS, 50 mg β-carotene on alternative days for 2.1 years had no effect on incidence of MI, stroke, or CVD mortality during 4.1 years of follow-up [94]. Similarly, the WAC Study reported that 50 mg β-carotene on alternate days had no effect on the combined outcome of MI, stroke, coronary revascularization, or CVD death among 8171 female health professionals [89]. However, analyses of ATBC trial revealed significant adverse effects of 20 mg/day β-carotene on the risk of fatal coronary event, a post-trial first-ever nonfatal MI and even lung cancer [82,100,101].

#### 5.4.4. Flaws of Randomized Controlled Trials

Overall the results of antioxidant supplement randomized clinical trials were disappointing. The null or adverse results did not support long-term use of dietary antioxidant supplement for CVD prevention. Although the underlying mechanisms for the null or adverse effects are still not well known, some design points are worth discussing and improvement in future studies. In terms of supplement dose, the non-linear relationships between antioxidant intake and disease risks indicate that a cut-off value exists for optimal health for some antioxidants. High dose of antioxidant intake may result in toxicity to human bodies [102]. Most of the antioxidants such as vitamin E, carotenoids, and uric acid can play a role as oxidants *in vivo* at their high concentrations. In addition, competitive inhibition may exist with a high dose of a single antioxidant. This was confirmed by the ATBC study, in which after a 6.7-year supplementation of 20 mg/day synthetic all-*trans* β-carotene, men in the supplement group had a significant decreased lutein level compared with men in the placebo group [73]. Different physical conditions and family CVD history of the participants might also contribute to the diverse results seen in different studies. As shown in the PHS-II, vitamin E supplement had significant interaction with parental history of MI <60 years, with a lower CVD risk seen in persons with a family history [90]. As discussed earlier, different genotype should be another consideration in the future studies.

### 5.5. Dietary Polyphenols

Polyphenols are widely distributed in the human diet, mainly derived from plant foods such as fruits, vegetables, nuts, seeds, tea, red wine, and cocoa. Polyphenols are characterized by having at least one aromatic ring with one or more hydroxyl groups attached and have been reported to have more than 8000 structures. These phytochemicals are plant secondary metabolites, the substances that have little or no role in photosynthesis or growth, but may accumulate in very high concentrations. The main role of polyphenols in plants is that they are involved in defense against infection and provide protective effects to the plants against external stimuli such as ultraviolet radiation, pathogens, and physical damage [9]. Polyphenols can be classified into two groups, the flavonoids and non-flavonoids, according to their structures. Flavonoids are the major constituents of polyphenols. Their basic structure consists of 15 carbons arranged in 3 rings—2 aromatic rings and a three-carbon bridge. Over 4000 naturally occurring flavonoids have been identified. Six classes of flavonoids are commonly found in human diet (flavan-3-ols (e.g., catechin, epicatechin), flavonols (e.g., quercetin, myricetin, kaempferol), anthocyanidins (e.g., cyanidin, delphinidin), flavones (e.g., apigenin, diosmin), flavanones (e.g., naringenin, hesperetin), and isoflavones (e.g., genistein, daidzein)). Non-flavonoid compounds contain an aromatic ring with one or more hydroxyl groups. This group includes stilben (e.g., resveratrol), phenolic acids (e.g., gallic acid), saponin (e.g., ginsenoside), and other polyphenols like curcumin and proanthocyanidin (or tannins) which are the polymers of flavan-3-ols [103].

#### 5.5.1. Estimation of Polyphenol Intake

Accurate estimation of daily intake of polyphenol is critical for investigation of the association between polyphenol and CVD risk. Before 2003, when United States Department of Agriculture (USDA) released a database on the flavonoid content of selected foods, only 4 of the 6 major classes of flavonoids were available for study, including flavonols, flavones, flavan-3-ols, and isoflavones. The flavonoid data used in these studies were based on the analyses originally conducted in Netherlands [104] and later were supplemented with additional food items. Anthocyanidin contents in foods were still not available which later were found to be an important group of flavonoids that was inversely associated with CVD incidence [75]. In 2003, USDA released the first flavonoid databases consisting of 26 flavonoids from five classes: flavonols, flavones, flavanones, flavan-3-ols, and anthocyanidins [105]. This database was recently updated in 2013. In 2004, proanthocyanidin contents of selected foods were also released [106]. A database of isoflavone content in 128 foods has been available since 1999 [107]. Other recent flavonoid databases such as the Phenol Explorer [108] also provide extensive data for research use. These databases contain the most recent publicly available data on flavonoid content of foods. Although few food processes and mixture of foods were considered in those databases, which have been shown to affect the bioavailability of polyphenols [109], combining the 3 USDA databases provides a more complete and reliable method for dietary flavonoid intake estimation.

#### 5.5.2. Prospective Studies

So far, only a few cohort studies have examined flavonoids and CVD risk, with three studies utilizing the more complete and reliable databases from USDA for flavonoid intake estimation. The properties of these studies were also summarized in Table 3 [73,74,75,76,77]. Of the five studies identified, two studies used food tables for dietary flavonoid estimation. The WHS was conducted among 38,445 female US health professionals aged ≥45 years in 1992. The cohort was followed up for a mean period of 6.9 years. After adjustment for covariates, no significant linear trend was observed across quintiles of total flavonoid intake for CVD or important vascular events [73]. The other is the NHS in which 66,360 female nurses in the US aged 33–55 years were included. Similarly, no association was observed between flavonol or flavone intake and risk of nonfatal MI or fatal CHD [74].

The other three studies were conducted after the release of the USDA flavonoids databases. The IWHS was conducted among 34,489 postmenopausal women who were free of CVD. After adjustment for covariates, a significant inverse association was found between dietary anthocyanidins intake and CHD (RR: 0.88; 95% CI: 0.78, 0.99) and CVD (RR: 0.91; 95% CI: 0.83, 0.99) when comparing any intake with no intake; between dietary flavanones intake and CHD (RR: 0.78; 95% CI: 0.65, 0.94) when comparing the highest quintile with the lowest quintile [75]. In the KIHD study, after multivariate adjustment, men in the highest quartile of flavonol and flavan-3-ol intakes had a 45% (RR: 0.55; 95% CI: 0.31, 0.99) and a 41% (RR: 0.59; 95% CI: 0.30, 1.14) lower risk for ischaemic stroke, respectively, as compared with the men in the lowest quartiles. Also, men in the highest quartile of flavanone intake had a 46% (RR: 0.54; 95% CI: 0.32, 0.92) lower risk for CVD death [76]. Recently, a subset population of 98,469 participants from Cancer Prevention Study II Nutrition Cohort was involved in the analysis of the association between flavonoid intake and CVD mortality after a follow-up period of 7 years. A 152-item modified Willet FFQ was used to collect dietary data. Flavonoid values were derived from the three USDA databases. After adjustment for many confounders, men and women in the highest quintile of total flavonoid intake had an 18% lower risk of fatal CVD (RR: 0.82; 95% CI: 0.73, 0.92) compared with participants in the lowest quintile [77].

#### 5.5.3. Randomized Controlled Trials of Polyphenol and CVD Risk Factors

Observational studies suggest a protective role of polyphenols against CVD. Although there are no clinical trials focusing on CVD incidence or mortality as outcome, several mechanisms of action have been proposed and explored in clinical studies.

*Antioxidant effects*. Polyphenols are one of the most potent antioxidants due to its structural characteristics. Polyphenols have been suggested to protect oxidative damage to big molecules including lipids, lipoproteins, and DNA [110]. Although antioxidant capacity of polyphenols has long been proven in numerous *in vitro* studies [111], clinical trials reported mixed results on the effect of polyphenols in modulating redox status *in vivo*. There have been several biomarkers developed to reflect redox status *in vivo*, such as plasma TAC, malondialdehyde (MDA), oxLDL, and, F_2_-isoprostanes.

Plasma TAC has been shown to reflect antioxidant intake status [112] and inversely associated with disease conditions [56] in some studies. Acute studies that investigated the effects of polyphenol-rich foods on plasma TAC consistently found significant elevations in plasma TAC several hours after consumption of fruits and vegetables [113], tea [114,115], red wine [116], chocolate [117] and, nuts [118]. The acute ingestion design is a reliable model to test the contribution of polyphenol-rich foods and beverages on *in vivo* antioxidant status, because this type of study is free from interferences by other food intake. One of the studies aimed to examine whether the nonalcoholic component of wine increases plasma TAC and whether such an effect is associated with the presence of phenolic compounds in plasma [116]. Compared to tap water and alcohol-free white wine groups, ingestion of alcohol-free red wine caused significant increases in plasma TAC values and polyphenol concentrations 50 min after ingestion [116]. Similarly, a dose-dependent manner of the acute effects of green tea ingestion on plasma TAC was demonstrated in another randomized crossover study [119]. In these studies, the increase in plasma TAC was attributed to the polyphenols present in these foods and beverages. However, other acute feeding studies found the increase in plasma TAC was paralleled an increase in uric acid concentrations [113,120,121]. Lotito and Frei [122] investigated plasma TAC, vitamin C, and uric acid changes after consumption of five apples and found that uric acid contributed to the increase in plasma TAC. In this study, plain bagels and water of comparable carbohydrate content and mass of five apples were used as a flavonoid-free control; a decrease in plasma TAC followed after bagel consumption, but a significant increase in plasma TAC and uric acid was observed after apple and fructose drink consumption. Subsequently, Lotito and Frei [122] reviewed numerous studies on this subject and pointed out that it was uric acid rather than polyphenols that accounted for the primary increase in plasma TAC, because uric acid had much higher concentration than polyphenols. However, the increased plasma TAC after tea consumption, which does not contain fructose, could not be explained by this hypothesized mechanism. On the other hand, chronic feeding studies generated inconclusive results in the effect of feeding polyphenol-rich food in modulating plasma TAC [123,124,125]. In a study that examined both acute and chronic effects of walnuts consumption on antioxidant status *in vivo* [123], 19-week walnut consumption did not significantly change the plasma antioxidant capacity of healthy old adults. The null results may be attributed to the weak control of other polyphenol-rich foods such as fruits and vegetables, although other nuts consumption was refrained during this period.

F_2_-isoprostanes, produced *in vivo* from arachidonic acid primarily through a non-enzymatic process of lipid peroxidation in cell membranes and LDL particles are currently regarded as the most reliable biomarkers of lipid peroxidation *in vivo* [126]. A decrease in urinary F_2_-isoprostanes was found after consumption of red wine and white wine [127], soy protein isolate [128], cherry juice [129], and almonds [130]. OxLDL is another biomarker of oxidative stress because various components of LDL can be oxidized including apoB, lipids, cholesterol, unsaturated fatty acids [131]. A decrease in oxLDL was observed after consumption of grape juice [132], green tea [133], and cocoa drink [134]. Other assays of oxidative stress such as thiobarbituric acid reactive substances (TBARS) and the *ex vivo* oxLDL were not discussed. Recently, the European Food Safety Authority disapproved the use of TBARS and the *ex vivo* oxidation lag time of LDL as biomarkers for lipid peroxidation [135]. TBARS assay has been frequently criticized for its lack of specificity for the reason that besides MDA, TBA also reacts with other aldehydes and non-aldehyde compounds. Besides antioxidant property, other functions of polyphenols were proposed by a number of studies and were reviewed below.

*Endothelial function improving effects*. Endothelial dysfunction is an integral component of atherosclerosis and is an independent predictor of cardiovascular risk. Dysfunction of the endothelium can be measured by flow-mediated dilation (FMD) of the brachial artery in human. Both acute and chronic feeding studies found an improvement on FMD after polyphenol-rich foods or polyphenol extracts consumption both in healthy individuals and coronary patients. An acute increase in FMD was found after consumption of polyphenol-rich foods such as red wine [136], chocolate, and cocoa [137,138]. In a randomized, single blind (operator), sham procedure-controlled, crossover design study, healthy participants took green tea, caffeine, or hot water after at least 8-h fasting. FMD increased significantly after tea consumption whereas no significant changes were seen following intake of caffeine or hot water [139]. Tea [140], chocolate or cocoa [141,142] have been well established for their protective function in FMD among heart disease patients or high risk population. Few studies investigated the effects of polyphenol extracts on FMD. A recently reported meta-analysis on the effect of oral isoﬂavone supplementation on vascular endothelial function in postmenopausal women [143] showed that oral supplementation of isoﬂavone had no influence on FMD if the age-adjusted baseline FMD was >5.2%. This study suggested that oral isoﬂavone supplementation does not improve endothelial function in postmenopausal women with high baseline FMD levels but leads to signiﬁcant improvement in women with low baseline FMD levels. In a randomized, double-blind, placebo-controlled crossover trial that examined grape seed supplement and FMD, adults with coronary disease or at least one cardiac risk factor receiving muscadine grape seed supplementation (1300 mg/day) for 4 weeks showed no evidence of improved FMD compared with people in the placebo group [144]. Due to the limited number of studies that investigated polyphenol supplements on FMD, no conclusion could be drawn at present in terms of their favorable effect on endothelial function.

*Effects on lipemia*. Significant reductions in LDL, apo B, lipoprotein (a), or increase in HDL and apo A1 were observed after consumption of tea [145], cocoa [146], and soy protein [147,148]. In a blinded randomized crossover study with 15 mildly hypercholesterolemic adults, tea, and other beverages were included in a carefully controlled weight-maintaining diet. After 3 weeks, 5 servings/day of tea reduced 6.5% total cholesterol, 11.1% LDL cholesterol (LDL-C), 5% apo B and 16.4% lipoprotein (a) compared with the placebo with added caffeine [145]. A randomized crossover trial with 22 young, healthy, normolipidemic subjects, who consumed diets providing 56 or 2 mg isoflavones/day for 17 days each, showed that plasma HDL-C and apo AI concentrations were elevated by 4% and 6%, respectively, after the high-isoflavone diet compared with the low-isoflavone diet [148]. However, in a meta-analysis of 10 clinical trials, soy proteins rather than soy-associated isoflavones were shown to effectively reduce LDL-C and increase HDL-C [149]. Future studies using isoflavones as a supplement are needed to establish the association between isoflavones and lipemia.

*Anti-inflammatory effects*. Although there have been numerous cell and animal studies on the effects of polyphenols on inflammation [150], only a few clinical studies have been reported. Recently, an acute study found that strawberry beverages signiﬁcantly attenuated the postprandial inﬂammatory response as measured by high-sensitivity CRP and IL-6 induced by a high-carbohydrate, moderate-fat meal [151]. In chronic studies, anti-inflammatory effect of polyphenols was also observed. A randomized, crossover investigation with 48 patients with peripheral arterial disease showed that 28-day supplementation of orange and black currant juice (500 mL/day) decreased CRP by 11% and ﬁbrinogen by 3% [152]. In a double-blind placebo controlled trial with healthy, non-smoking men, consumption of black tea for 6 weeks reduced the number of monocyte-platelet aggregates, neutrophil-platelet aggregates, total leukocyte-platelet aggregates, and CRP compared with the placebo [153]. Also, circulating VCAM-1concentrations were reduced after the 6-week administration of isoflavones [154]. Null results were also reported in some studies [155,156]. In a randomized control study, the participants consumed one of four strictly controlled isocaloric diets containing either rich or poor in vegetables, berries and apple and rich either in linoleic acid or oleic acid [156] for six weeks. In this study, 90% kcal of daily diet was prepared by the research group in order to strictly control nutrient intake. However, 6 weeks of intervention did not change the ICAM-1 or CRP levels among the subjects. The limited anti-inflammatory effects of polyphenols observed in these human studies may be attributable to the fact that they were conducted on healthy subjects.

*Other effects*. Gene-regulating effects of polyphenols has been proposed and investigated in recent years. Cell and animal studies have suggested that polyphenols could elevate the activities of antioxidant enzymes [157] by activating the antioxidant response element upstream of genes that are involved in antioxidation and detoxification [158], or phosphorylating nuclear factor erythroid 2-related factor 2 [159]. Regulation of antioxidant enzyme system and signaling pathway by polyphenols may explain their protective effect against oxidative stress and inflammation and CVD, as polyphenols have remarkably low bioavailability and are converted to diverse metabolites *in vivo*. Another new hypothesis has been put forth that polyphenols may modulate gut microbiota. Human gut microbiota composition has been found to be associated with obesity and inflammation [160,161]. Polyphenols are known to have antimicrobial properties which may be linked to their anti-inflammatory effect [162]. These hypotheses need to be further tested in future clinical studies.

### 5.6. Dietary TAC

Given the evidence that single antioxidant supplements showed no beneficial effects on preventing CVD, as well as the fact that diet high in antioxidants such as fruits, vegetables, and tea has been widely reported to have beneficial health effects, dietary TAC that considers all the antioxidants present in diet and the synergistic effects between them thus are drawing increasing attention. Dietary TAC has been found to be positively associated with several diet quality scores [163], as well as typical individual antioxidants, indicating dietary TAC represents dietary quality and antioxidant status in these study populations [164].

#### 5.6.1. Estimation of Dietary TAC

Estimating dietary TAC is the first step in investigating its relationship to chronic diseases. Two types of methods have been developed to assess dietary TAC: experimental method and theoretical method. Experimental method utilizes a food-based TAC database to directly calculate TAC in diet by adding the TAC value of each food item together. In such a database, commonly consumed food items were measured for TAC values by one of the established TAC assays such as Trolox equivalent antioxidant capacity (TEAC), and oxygen radical absorbance capacity (ORAC). Several countries have developed their own dietary TAC databases [165,166,167,168,169]. The theoretical method combines a nutrient-based TAC database and a nutrient composition database of food items to calculate dietary TAC. In a recent study, 44 single antioxidants were measured for the TAC values [170], dietary TAC of each food item theoretically calculated was significantly correlated with the value measured from experimental method. One of the advantages of the theoretical method is that TAC supplements could also be estimated. By using this method, Yang *et al.* [171] estimated dietary TAC from both diet and supplements from NHANES 2001–2002 and found dietary TAC was higher in women, older people, Caucasians, people with higher income levels and higher exercise levels.

#### 5.6.2. Epidemiological Studies

Recently, three large cohort studies investigated the associations between dietary TAC and stroke and MI [78,79,80]. In the Italian cohort of the EPIC study, TAC values of around 150 food items were measured by TEAC assay and dietary TAC was calculated. Men and women (*n* = 41,620) free of stroke and MI were followed for 7.9 years on average. Dietary TAC intake was inversely associated with ischemic stroke cases (hazard ratio: 0.41; 95% CI: 0.23, 0.74) but not hemorrhagic stroke. Of vitamin C, vitamin E, and β-carotene, vitamin C intake attributed most to the inverse association [78]. The Swedish Mammography Cohort involved 31,035 women free of CVD and 5680 women with CVD history at baseline. After a 12-year follow-up, dietary TAC was inversely associated with total stroke in the CVD-free cohort (*p*_trend_ = 0.04), but not in CVD history cohort. In the CVD history cohort, higher TAC was associated with a lower risk for hemorrhagic stroke [79]. Another study of the Swedish Mammography Cohort involved 32,561 women aged 49–83 years old free of CVD at baseline. An ORAC food database was used to calculate dietary TAC. After a follow up of 10 years, women in the highest quintile of dietary TAC intake had a 20% lower risk of MI (RR: 0.80; 95% CI: 0.67–0.97) after adjustment for other risk factors [80].

Several cross-sectional studies also reported inverse associations between dietary TAC and CRP [172], oxidative stress biomarkers [173], diabetes biomarkers [174], and metabolic markers [173,175]. Two Italian intervention studies found intake of diet high in TAC for two weeks significantly decreased inflammatory biomarkers [176] and improved endothelial function measured by FMD [177] among the elderly. However, due to the criticism that flavonoids may not work as antioxidants *in vivo* and unidentified mechanisms may contribute to their protective effects, USDA has recently withdrawn the ORAC database that was previously available providing TAC values of selected food items, to avoid misuse by food and supplement companies. Overall, epidemiological studies suggested an association between dietary TAC and CVD risk, although the evidence is still limited.

## 6. Conclusions

Although antioxidant supplement use was reported to have no effect or an adverse effect on cardiovascular events by several large randomized controlled trials, cohort studies still supported the protective effects of dietary antioxidants on preventing CVD. Besides antioxidant vitamins, polyphenols are a large group of compounds that exhibit high antioxidant capacity *in vitro* and cardioprotective effects *in vivo*. Although the *in vivo* effects of polyphenols may be beyond the scope of the topic-antioxidants of this review, considering the synergistic effect between antioxidants, dietary quality scores, and dietary TAC that considered the overall diet quality are still worth investigating with regard to the association between diet and CVD.
